# Healthcare burden and clinical outcomes of polypharmacy in older adults: a population-based cohort study in South Korea

**DOI:** 10.1186/s13690-025-01703-3

**Published:** 2025-08-25

**Authors:** Zhaoyan Piao, Kyung Sun Oh, Euna Han

**Affiliations:** 1 College of Pharmacy, Yonsei Institute of Pharmaceutical Sciences, Yonsei University, 162-1 Songdo-Dong, Yeonsu-Gu, Incheon, Republic of Korea; 2 College of Pharmacy, Yeungnam University, 280 Daehak-ro, Gyeongsan-si, Gyeongsangbuk-do, Republic of Korea

**Keywords:** Polypharmacy, Geriatrics, Multivariable linear regression analysis, Two-part model, Living alone

## Abstract

**Background:**

Polypharmacy is a prevalent issue in aging societies, with potential health and cost implications. This study evaluated the impact of polypharmacy on hospitalization rates and healthcare expenditure among individuals aged 65 years and older in South Korea.

**Methods:**

We analyzed integrated data from the 2012–2016 Korean National Health and Nutrition Examination Survey alongside administrative claims data from the National Health Insurance Service and Health Insurance Review and Assessment Service. Medical costs were calculated as total annual per capita expenses, encompassing outpatient visit, hospitalization, and drug costs. Key clinical outcomes included hospitalization and mortality. To evaluate the effects of polypharmacy on outpatient visits, medication costs, and total expenditures, we performed a multivariable linear regression analysis. A two-part model was used to estimate hospitalization costs.

**Results:**

The analysis included 3,297 participants. Polypharmacy was significantly associated with higher hospitalization risk (OR, 1.52; 95% CI, 1.28–1.81) and mortality (OR, 3.17; 95% CI, 1.19–8.44). Polypharmacy also corresponded with increased healthcare expenditures, particularly in total, outpatient visit, and medication costs, with the highest associations observed in individuals aged 65–79 and those living alone. Multivariable linear regression revealed a higher annual average total healthcare cost by 872,018 KRW, with outpatient and medication costs by 324,879 KRW and 536,721 KRW, respectively (*p* < 0.05) for polypharmacy group than the counterparts.

**Conclusion:**

This study demonstrates that polypharmacy among older adults is significantly associated with higher healthcare costs and risks of hospitalization and mortality. Findings suggest that integrated care models, incorporating medication reviews and tailored care plans, alongside community resources to support isolated seniors, may mitigate healthcare costs and improve health outcomes.

**Supplementary Information:**

The online version contains supplementary material available at 10.1186/s13690-025-01703-3.

**Table:** 

**Text box 1. Contributions to the literature**
• Evidence on the economic and clinical burden of polypharmacy among older adults in South Korea remains limited.
• This study shows that polypharmacy is linked to increased hospitalization, mortality, and healthcare costs, especially in those aged 65–79 and living alone.
• The findings support the development of integrated care models, including medication reviews and social support services, to reduce risks and spending.
• Results provide actionable insights for healthcare policymakers aiming to improve care coordination and reduce unnecessary medication use among the elderly.

## Background

South Korea is experiencing one of the most rapid demographic transitions toward an aging population worldwide. As of 2020, individuals aged 65 years and older comprised 15.7% of the total population [1], and projections indicate this proportion will increase to approximately 25.3% by 2030 [1]. This demographic shift is accompanied by a range of healthcare challenges, notably the rising incidence of polypharmacy among older adults [2]. 

The prevalence of chronic health conditions necessitates the use of multiple medications [3, 4]. In the Korean healthcare system, patients with multimorbidity such as hypertension, diabetes, and dyslipidemia often receive disease-specific care from multiple providers, leading to fragmented management [5]. This contributes to a higher prevalence of polypharmacy, the use of therapeutically duplicative medications, and increased regimen complexity, all of which can reduce medication adherence and lead to additional healthcare utilization and expenditure [5]. Previous research in Korea has shown that polypharmacy is more common among certain vulnerable groups, such as aged 80 and older and recipients of long-term care services. However, few studies have examined the risks of hospitalization and the associated medical and medication costs in patient groups with a high rate of polypharmacy [6, 7]. 

This study evaluated the association of polypharmacy with hospitalization rates and healthcare expenditure among individuals aged 65 years and older in South Korea. We utilized health insurance claims data integrated with the National Health and Nutrition Survey to conduct comprehensive analysis by combining objective medical utilization records with detailed health behavior and nutritional data for a representative sample of the population. We stratified the sample into younger seniors (aged 65 years) and individuals approximately 80 years of age to clarify the distinct effects of polypharmacy within these subgroups. Furthermore, we examined specific patterns of polypharmacy and healthcare utilization among older adults living alone, a demographic group recognized as vulnerable due to limited social support, economic hardship, or difficulty accessing essential services [8]. Our research provides evidence-based insights that can inform health promotion and cost-reduction strategies through optimized polypharmacy management, ultimately contributing to a sustainable healthcare system in the context of an ultra-aging society.

## Methods

### Data source

We utilized data from the Korea National Health and Nutrition Examination Survey (KNHANES) from 2012 to 2016, which were linked to administrative claims data from the Health Insurance Review and Assessment Service (HIRA), as well as from the National Health Insurance Service (NHIS) from 2012 to 2017. (Supplementary Fig. 1).

KNHANES [9] is a national survey conducted by the Korean Agency for Disease Control and Prevention that assesses the health and nutritional status of the Korean population through interviews and physical examination. All participants were selected based on demographic data such as gender, age, region, and type of residence in a stratified sample representative of the total Korean population [10]. The data collected include health behaviors, socioeconomic factors, clinical health indicators, and dietary habits [10]. 

The NHIS dataset includes beneficiary eligibility information, such as disability and mortality. Korea has a single health insurance claims database that is representative of the entire country, and claims data are generated when healthcare providers make insurance benefit claims to HIRA after providing medical care [11]. Therefore, HIRA provides representative health insurance data for Korea [11]. The combination of these datasets provides a solid foundation for assessing the health outcomes and healthcare utilization of the Korean population.

The study was approved by Inha University Hospital (2022-09-039-001). Informed consent was waived due to the study’s retrospective design.

### Study population

A total of 24,900 individuals participated in the KNHANES from 2012 to 2016. We excluded 18,457 respondents under 65 years of age, 1,397 who died or were hospitalized during the baseline survey, and 980 who did not have any outpatient medications or were prescribed for less than 30 days. After excluding another 769 individuals with missing data, 3,297 older adults were included in the final analysis. Claims data (2012–2017) were linked for 3,279 individuals to define polypharmacy and outcomes, with no missing values.

### Definitions and measurement

#### Polypharmacy

Polypharmacy was defined as more than 30 prescription days with five or more distinct prescriptions in six months (July–December, 2012–2016) [2, 12, 13]. We counted the number of drugs per respondent using the Korean national drug code from the WHO-Anatomical Therapeutic Chemical Classification System [14]. 

#### Medical costs and clinical outcomes

For medical expenses, we considered total annual per capita medical, outpatient visit, hospitalization, and medication cost. We also observed clinical outcomes, including hospitalization and mortality. The above results were observed in the year following polypharmacy measurement.

#### Covariates

This study included several covariates. Age was classified as 65–79 years and ≥ 80 years. To reflect the healthcare vulnerability of older adults living alone, we stratified the population into individuals living alone and those residing with others. Health lifestyles included not drinking excessively, walking more than 5 days per week, not smoking, and a body mass index (BMI; kg/m^2^) < 25. The Charlson Comorbidity Index (CCI) [15, 16] was classified as 0, 1, or ≥ 2, and area of residence was divided into metropolitan, urban, and rural depending on population density. Household income was divided into four quartiles: low, lower-middle, upper-middle, and high. Education level was divided into below high school and high school or higher. Health insurance was classified as either national health insurance or medical aid [2]. We also controlled disability, private health insurance and calendar year (survey year).

### Statistical analysis

We performed a multivariable linear regression analysis to examine the association of polypharmacy with medication cost, and total expenses. We used a two-part model to adjust the probability of hospitalization in predicting the hospitalization expenditure: firstly, a probability model was used to estimate the probability of being hospitalized in a one-year period; secondly, the same covariates with gamma distribution and log link were added to the model to estimate the cost of hospitalization conditional on incurring positive costs [17]. In addition, we performed a multivariable logistic regression analysis to examine whether polypharmacy is a risk factor for hospitalization or death. All analyses were conducted using SAS 9.4, with statistical significance defined as a two-sided p-value of less than 0.05.

## Results

The study included 3,297 participants, 86.78% of whom were between 65 and 79 years of age. Female participants comprised 54.87% of the sample. The majority of the sample (78.56%) lived with others, and 61.15% had a BMI < 25. Regarding health behaviors, 43.25% of the participants walked five or more days a week, 40.76% were non-smokers, and 96.97% did not consume extensive alcohol. In terms of area of residence, only 23.14% lived in rural areas. Nearly half of the sample (47.32%) lived in low-income households, with smaller proportions in the lower-middle (28.18%), upper-middle (14.38%), and upper (10.13%) income groups. Almost all respondents (93.72%) were enrolled in national health insurance, while 65.51% were enrolled in private health insurance. Regarding education level, 26.33% had completed high school or higher. The CCI showed that 17.02% of the participants had two or more comorbidities. (Table 1)

**Table 1:** Baseline characteristics

Variables	All (*n* = 3,297)	
	*N*	%
**Age (years)**		
65–79	2861	86.78
**Sex**		
Female	1809	54.87
**Living alone**		
No	2590	78.56
**BMI**		
< 25	2016	61.15
**Walking days per week**		
≥ 5	1426	43.25
**Ever smoked**		
No	1344	40.76
**Drink**		
Non-excessive	3197	96.97
**Residential area**		
Metropolitan	1501	45.53
Urban	1033	31.33
Rural	763	23.14
**Income quartile (household)**		
Low	1560	47.32
Lower-middle	929	28.18
Upper-middle	474	14.38
High	334	10.13
**Health insurance type**		
National health insurance	3090	93.72
**Private medical insurance**		
Yes	2160	65.51
**Health screening within 2 years**		
Yes	1064	32.27
**Cancer screening within 2 years**		
Yes	1214	36.82
**Education level**		
High school and above	868	26.33
**Charlson comorbidity index**		
0	2205	66.88
1	531	16.11
≥ 2	561	17.02
**Disability**		
Yes	479	14.53
**Usual perception of stress**		
Yes	626	18.99
**Calendar year**		
2012	689	20.90
2013	565	17.14
2014	610	18.50
2015	665	20.17
2016	768	23.29

**Fig. 1:**
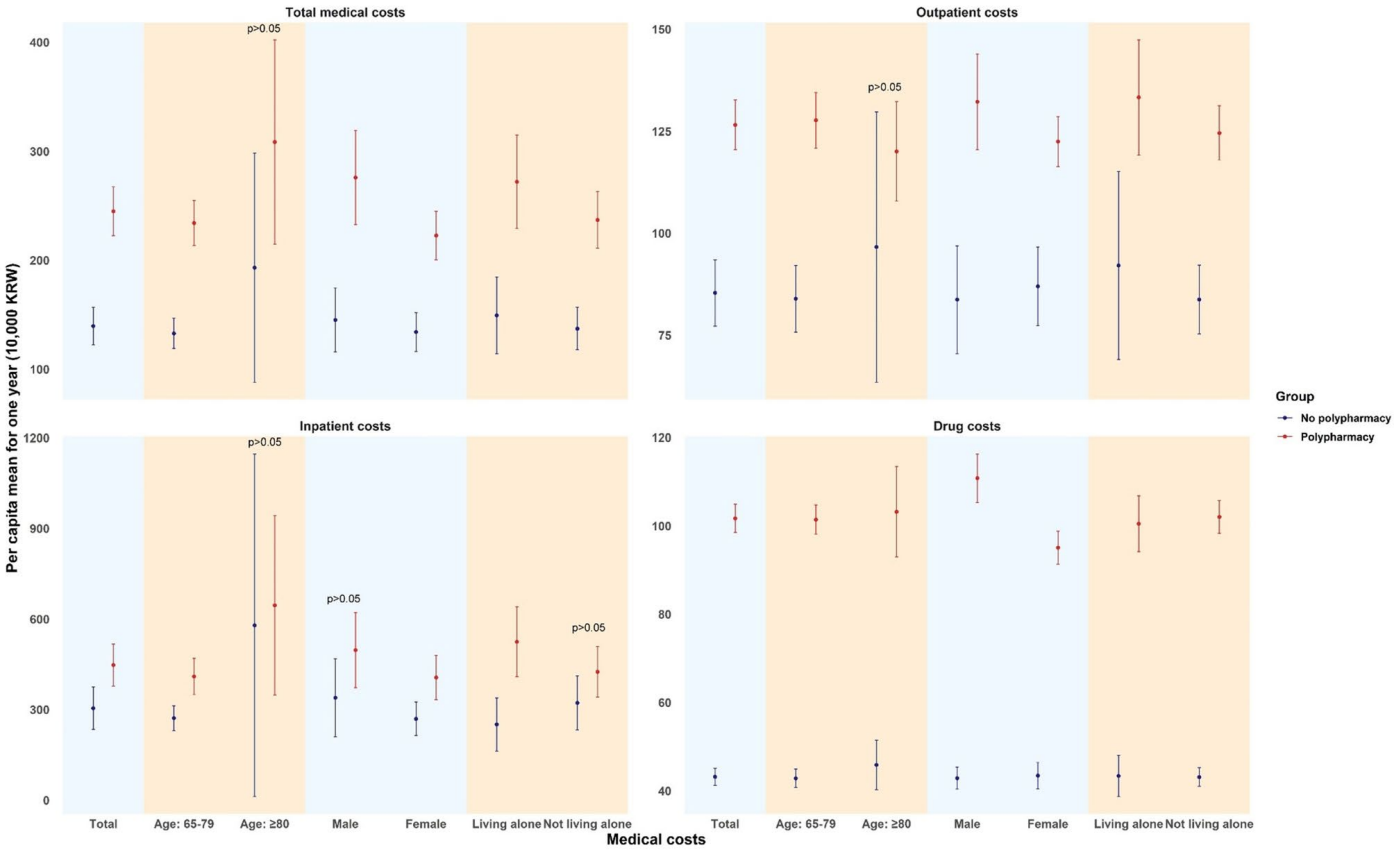
Hospitalization and mortality rates by polypharmacy

Figure 1 illustrates the hospitalization and mortality rates per 10,000 population and compares them based on polypharmacy use. Overall, hospitalization and mortality rates were significantly higher among people who used polypharmacy compared with those that did not. The effects of polypharmacy on hospitalization and mortality were more pronounced among individuals who were male, aged 80 years and over, and did not live alone, although the differences were smaller among those living alone.

Table 2 shows a significant association between polypharmacy and increased risk of hospitalization and death in total sample and subgroups of older adults. For the whole sample, polypharmacy was associated with a high risk of hospitalization (OR, 1.52; 95% CI, 1.28–1.81) and death (OR, 3.17; 95% CI, 1.19–8.44). Both the risks of hospitalization (OR, 1.48; 95% CI, 1.23–1.79) and death (OR, 3.72; 95% CI, 1.06–13.05) were higher for polypharmacy group in those aged 65–79 years, whereas only hospitalization risk was higher for polypharmacy group in those aged 80 years and older (OR, 1.78; 95% CI, 1.06–2.99). By gender, the risk of hospitalization for polypharmacy group was significantly higher only among men (OR, 68; 95% CI, 1.30–2.17). For people who did not live alone, polypharmacy was associated with a 1.69 times higher risk of hospitalization (95% CI, 1.38–2.06) and 4.08 times higher risk of death (95% CI, 1.18–14.12).

**Table 2:** Association between polypharmacy and clinical outcomes in older adults: multivariable logistic regression

Sample	Odds Ratio (OR) (95% Confidence Interval)	
	Hospitalization	Mortality
Total (*n* = 3,297)	1.52(1.28–1.81) *	3.17(1.19–8.44) *
65–79 years old (*n* = 2,861)	1.48(1.23–1.79) *	3.72(1.06–13.05) *
≥ 80 years old (*n* = 436)	1.78(1.06–2.99) *	1.90(0.37–9.68)
Males (*n* = 1,488)	1.68(1.30–2.17) *	2.91(0.80–10.59)
Females (*n* = 1,809)	1.40(1.10–1.79) *	3.23(0.70–14.94)
Living alone (*n* = 707)	1.05(0.72–1.53)	1.92(0.33–11.12)
Not living alone (*n* = 2,590)	1.69(1.38–2.06) *	4.08(1.18–14.12) *

*Notes.* Models controlling for age (years), sex, residential areas, income quartile (household), health insurance type, having private medical insurance, health screening in 2 years, cancer screening in 2 years, education level, Charlson comorbidity index, disability, BMI, living alone, walking days per week, having ever smoked, non-excessive drink, and calendar year. *p < 0.05

**Fig. 2:**
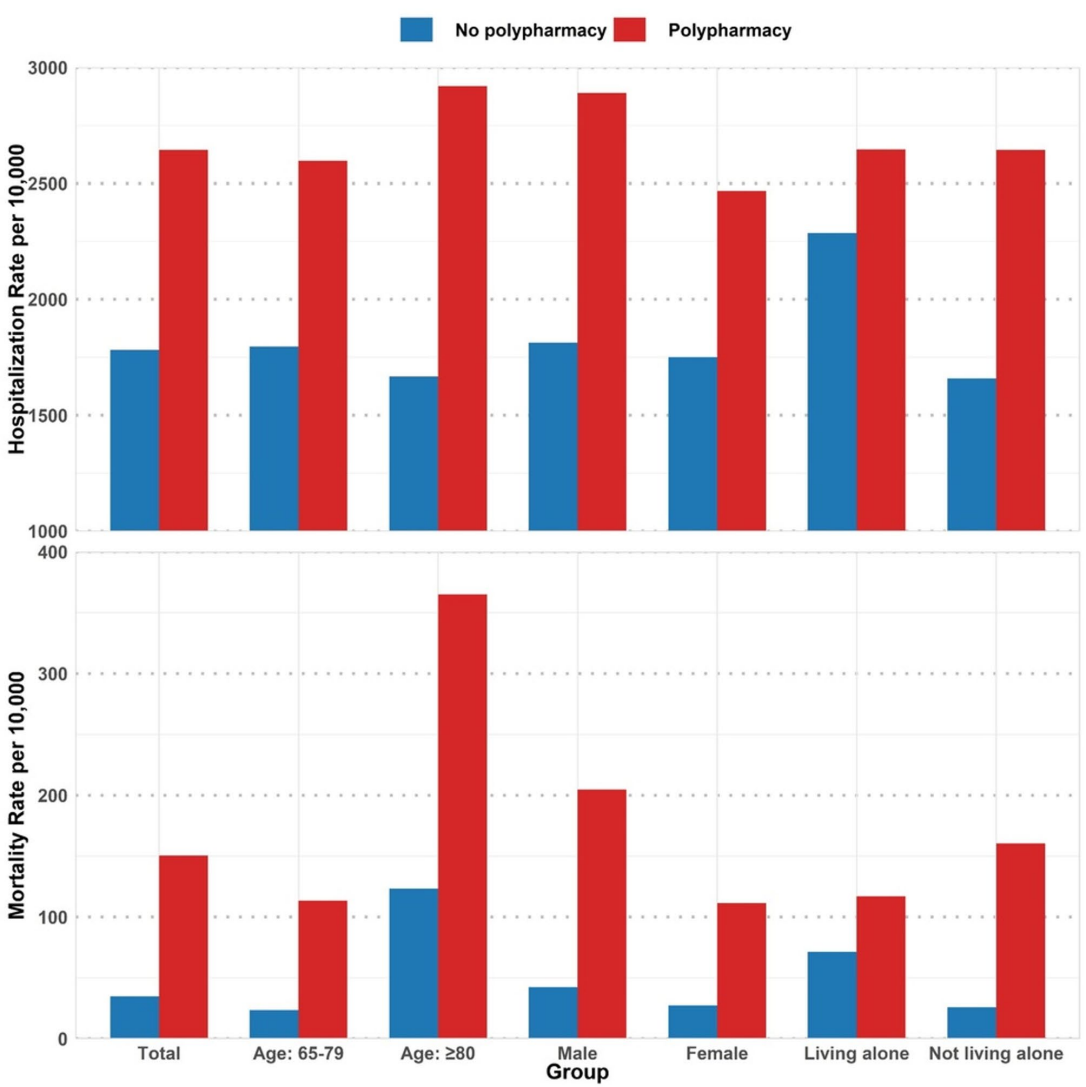
Medical costs by polypharmacy

As shown in Fig. 2, polypharmacy was statistically significantly associated with a higher average annual healthcare cost across subgroups. Total medical costs were higher for those with polypharmacy, especially for men, those aged ≥ 80 years, and those not living alone (*p* < 0.05). Outpatient and drug costs were also statistically significantly higher for most subgroups among those with polypharmacy; however, the differences for outpatient and inpatient costs was not statistically significant for those aged 80 years or older (*p* > 0.05). Inpatient costs were significantly higher for those with polypharmacy than without among those aged 65–79 years, women, and those living alone. Drug costs in the polypharmacy group were significantly higher in all subgroups of the study sample. Overall, polypharmacy was associated with increased healthcare costs, especially among respondents in the 65–79 age group and those who lived alone.

Table 3 shows the association between polypharmacy and annual medical costs. In the multivariable linear regression, polypharmacy was significantly associated with increased total healthcare, outpatient, and drug costs. Total medical costs were 872,018 KRW per year higher (*p* < 0.05), outpatient costs were 324,879 KRW (*p* < 0.05) higher, and drug costs were 536,721 KRW (*p* < 0.05) higher for those with polypharmacy than those without. The differences in total and outpatient costs between the polypharmacy and non-polypharmacy respondents were statistically significant in all subgroups, except in people aged 80 years or older. In the two-part model examining hospitalization probability, polypharmacy was associated with a significant increase in hospitalization risk. Regarding total hospitalization costs, the total inpatient cost for the polypharmacy group was 1,282,226 KRW without adjusting for the probability of hospitalization (*p* < 0.05.) After adjusting for the probability of hospitalization, the total hospitalization costs were 538,534 KRW higher in the polypharmacy group than in the non-polypharmacy group (non-significant). In the 65–79 years old age group, the incremental total hospitalization costs adjusting the probability of hospitalization by polypharmacy was 522,926 KRW (*p* < 0.05).

**Table 3:** The association between polypharmacy and healthcare cost (KRW)

		Regression estimate for polypharmacy (standard error)			
	Multivariable regression				Two-part model
Reference Non-polypharmacy Comparison Polypharmacy	Total cost^1^	Outpatient costs^1^	Drug costs ^1^		Inpatient costs^2^
Total (*n* = 3,297)	872,018 (153,868) *	324,879 (51,512) *	536,721 (20,589) *		1,282,226 (569,015) *
65–79 (*n* = 2,861)	847,661 (136,103) *	359,304 (54,547) *	534,199 (21,104) *		1,340,837 (447,457) *
≥ 80 (*n* = 436)	825,784 (771,406)	138,873 (156,864)	529,577 (74,149) *		1,056,337 (3,288,392)
Male (*n* = 1,488)	1,034,734 (278,886) *	390,089 (91,796) *	634,259 (32,586) *		1,213,485 (1,008,922)
Female (*n* = 1,809)	703,429 (160,331) *	270,219 (55,606) *	451,030 (26,085) *		1,257,406 (591,987) *
Living alone (*n* = 707)	1,080,115 (314,014) *	355,408 (133,679) *	494,303 (43,979) *		2,851,996 (897,463) *
Not living alone (*n* = 2,590)	819,306 (176,264) *	327,679 (54,811) *	548,063 (23,362) *		857,487 (693,495) *

*Notes.* The two-part model for inpatient costs included 748 people who had hospitalization experience, of whom 641 were 65–79 years old, 107 were ≥ 80 years old, 354 were male, 394 were female, 177 lived alone, and 571 did not live alone. Regression estimates for the two-part model was adjusted for the probability of hospitalization. All models controlled for age (years), sex, residential areas, income quartile (household), health insurance type, having private medical insurance, health screening in 2 years, cancer screening in 2 years, education level, Charlson comorbidity index, disability, BMI, living alone, walking days per week, having ever smoked, non-excessive drinking, and calendar year. Standard errors are in brackets. *p < 0.05

## Discussion

This study demonstrated that polypharmacy among older adults was significantly associated with increased healthcare costs, including medication expenses, as well as heightened hospitalization risks. Notably, we found a significant association between polypharmacy and both hospitalization and mortality rates, particularly among those aged 65–79 years. Furthermore, older adults living alone exhibited particularly high hospitalization rates when not engaging in polypharmacy compared to their counterparts in other groups.

The association between polypharmacy and rising healthcare expenditures has been corroborated by several previous studies [4, 18, 19]. For example, among older adults in the United States with cardiovascular disease prescribed five or more medications, average pharmacy-related expenditure was $1,286, which was significantly higher than the expected average of $488 for those not engaged in polypharmacy [4]. This discrepancy underscores the economic burden that polypharmacy imposes on individuals and the healthcare system. Similarly, research conducted in Sweden identified increased polypharmacy prescriptions as a primary factor contributing to escalating medication-related healthcare costs [18]. In our study, older adults engaged in polypharmacy were found to incur annual healthcare expenses 872,018 KRW higher than their non-polypharmacy counterparts, aligning with existing literature and reinforcing the urgent need for effective management strategies to address polypharmacy in older adult patients.

Furthermore, we found that older adults living alone were particularly susceptible to high hospitalization rates in the non-polypharmacy group. The results indicated that among older adults living alone, the difference in hospitalization rates between those who used polypharmacy and those who did not was notably smaller compared to other demographic groups, whereas the mortality rate in the non-polypharmacy group was significantly higher. This phenomenon may be linked to economic independence and varying levels of social support experienced by older adults living alone [20]. Insufficient access to appropriate medication to treat chronic health conditions could lead to higher hospitalization rates among those not engaged in polypharmacy. Such findings align with the existing literature highlighting the adverse effects of social isolation on the health of older adults, suggesting that those who live alone may face barriers to accessing adequate healthcare and medication management [21–23]. 

This study, as a retrospective investigation of polypharmacy and healthcare utilization, has several limitations. Most notably, it does not account for medication-related issues, such as medication adherence, inappropriate medication prescribing, or the severity of underlying comorbidities. These unmeasured factors may contribute to residual confounding, and polypharmacy may reflect overall disease burden rather than act as a direct causal factor. Furthermore, important variables, such as the presence of socioeconomic support for older adults living alone, a potentially critical factor in healthcare outcomes, were not included in the analysis. Despite these limitations, the study also has several notable strengths. First, it leveraged objective and reliable data sources by integrating the nationally representative sample from KNHANES with health insurance claims data. This methodology provided robust insights into the relationship between polypharmacy, healthcare utilization, and associated expenditure. Second, the study reflected the growing demographic challenges related to an aging population, focusing specifically on older adults living alone. Analyzing polypharmacy’s association with hospitalization and healthcare costs in this vulnerable subgroup contributes to a broader understanding of the unique health risks faced by socially isolated older adults.

## Conclusions

This study elucidated the association of polypharmacy with healthcare utilization and medical costs among older adults, particularly within socially isolated populations. Given the increasing prevalence of polypharmacy among older adults, these findings emphasize the critical need for integrated care approaches that address both medical and social factors. Healthcare providers should prioritize providing routine medication assessments, identifying potentially inappropriate medication, and implementing individualized care plans. Furthermore, implementing tailored support services for socially isolated older adults, including improved access to community resources, may contribute to lowering healthcare expenditures.

## Supplementary Information

Below is the link to the electronic supplementary material.

Supplementary Material 1

## Data Availability

No datasets were generated or analysed during the current study.

## Abbreviations

KNHANES: Korean National Health and Nutrition Examination Survey
NHIS: National Health Insurance Service
HIRA: Health Insurance Review and Assessment Service
KRW: South Korean Won
