# Supplementary figures and images for: Healthcare burden and clinical outcomes of polypharmacy in older adults: a population-based cohort study in South Korea
Source: Arch Public Health. 2025 Aug 25;83:217. doi: 10.1186/s13690-025-01703-3 (PMC12376462; doi:10.1186/s13690-025-01703-3)

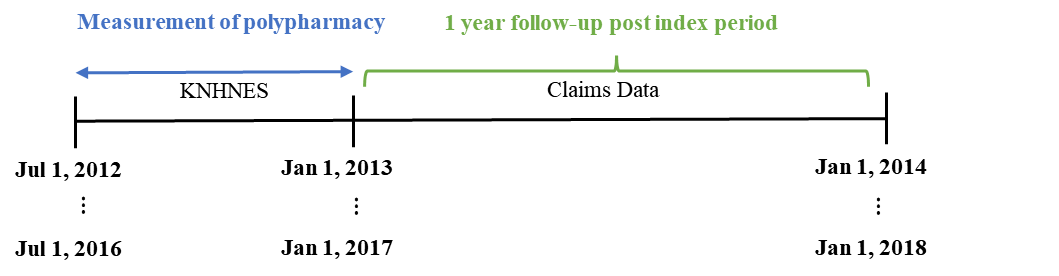
**Supplementary Figure 1.** Study design

Supplement: Supplementary file 1 — Supplementary Material 1 [file 13690_2025_1703_MOESM1_ESM.docx]
